# Mutator-Derived lncRNA Landscape: A Novel Insight Into the Genomic Instability of Prostate Cancer

**DOI:** 10.3389/fonc.2022.876531

**Published:** 2022-07-04

**Authors:** Liansha Tang, Wanjiang Li, Hang Xu, Xiaonan Zheng, Shi Qiu, Wenbo He, Qiang Wei, Jianzhong Ai, Lu Yang, Jiyan Liu

**Affiliations:** ^1^ Department of Biotherapy, West China Hospital, Sichuan University, Chengdu, China; ^2^ West China Medical School of Sichuan University, Chengdu, China; ^3^ Department of Radiology, West China Hospital of Sichuan University, Chengdu, China; ^4^ Department of Urology, Institute of Urology, West China Hospital of Sichuan University, Chengdu, China; ^5^ Institute of System Genetics, West China Hospital of Sichuan University, Chengdu, China

**Keywords:** long non-coding RNAs, genome instability, mutation phenotype, prostate cancer, biochemical recurrence

## Abstract

**Background:**

Increasing evidence has emerged to reveal the correlation between genomic instability and long non-coding RNAs (lncRNAs). The genomic instability-derived lncRNA landscape of prostate cancer (PCa) and its critical clinical implications remain to be understood.

**Methods:**

Patients diagnosed with PCa were recruited from The Cancer Genome Atlas (TCGA) program. Genomic instability-associated lncRNAs were identified by a mutator hypothesis-originated calculative approach. A signature (GILncSig) was derived from genomic instability-associated lncRNAs to classify PCa patients into high-risk and low-risk groups. The biochemical recurrence (BCR) model of a genomic instability-derived lncRNA signature (GILncSig) was established by Cox regression and stratified analysis in the train set. Then its prognostic value and association with clinical features were verified by Kaplan–Meier (K-M) analysis and receiver operating characteristic (ROC) curve in the test set and the total patient set. The regulatory network of transcription factors (TFs) and lncRNAs was established to evaluate TF–lncRNA interactions.

**Results:**

A total of 95 genomic instability-associated lncRNAs of PCa were identified. We constructed the GILncSig based on 10 lncRNAs with independent prognostic value. GILncSig separated patients into the high-risk (*n* = 121) group and the low-risk (*n* = 121) group in the train set. Patients with high GILncSig score suffered from more frequent BCR than those with low GILncSig score. The results were further validated in the test set, the whole TCGA cohort, and different subgroups stratified by age and Gleason score (GS). A high GILncSig risk score was significantly associated with a high mutation burden and a low critical gene expression (*PTEN* and *CDK12*) in PCa. The predictive performance of our BCR model based on GILncSig outperformed other existing BCR models of PCa based on lncRNAs. The GILncSig also showed a remarkable ability to predict BCR in the subgroup of patients with *TP53* mutation or wild type. Transcription factors, such as FOXA1, JUND, and SRF, were found to participate in the regulation of lncRNAs with prognostic value.

**Conclusion:**

In summary, we developed a prognostic signature of BCR based on genomic instability-associated lncRNAs for PCa, which may provide new insights into the epigenetic mechanism of BCR.

## Background

Prostate cancer (PCa) is one of the most highly prevalent genitourinary malignancies among men in developed countries and is regarded as the second major cause of cancer-related death (1). The 5-year relative survival rate of men with PCa is 98%, which may be attributed to the early test of prostate-specific antigen (PSA) combined with prostate needle biopsy and advanced therapy strategies (2). However, patients with distant PCa only have a 5-year survival rate of 31% (1), and 20%–53% of men would eventually experience biochemical recurrence (BCR) after long-term follow-up (3). Established predictive biomarkers, such as biopsy core involvement percentage and Gleason grade, are restricted to clinicopathological features and could not explain genomic heterogeneity that emphasizes the biological complexity of PCa (4). Notably, tumor heterogeneity exists across subtypes of PCa, and multiple genetic mutations can influence tumor progression and tumor response to medical treatments (5). Therefore, it is necessary to propose specific and sensitive molecular indices to predict the clinical outcomes of PCa more accurately (6, 7).

Genomic instability, including DNA repair defects and chromosome instability (CIN), is reported to be the driving force of genetic heterogeneity (8, 9). It is regarded as a fundamental hallmark of human cancer and is associated with tumor pathogenesis and progression (10). In PCa, copy number aberrations and translocations, as the structural genome rearrangements, are a critical mechanism of tumorigenesis (11, 12). Chung et al. (13) analyzed the comprehensive genomic profiling of 1,660 primary and 1,816 metastatic PCa specimens to find the signatures of genomic instability. They found that *TP53* (44%) and *PTEN* (32%) were the frequently altered genes. Tam et al. (14) demonstrated that transcription-related aberration and regulation might be implicated in genomic instability, suggesting the potential of molecular features in genomic instability quantitative evaluation. However, the cause of genomic instability in PCa has not been fully understood.

Long non-coding RNAs (lncRNAs) broadly refer to transcripts without protein-coding potentiality (15, 16). More than 200 nucleotides in length are arbitrarily considered to become a practical cutoff that differentiates lncRNAs from short ncRNAs [microRNAs (miRNAs)] (17). Recently, lncRNAs have become a research hotspot in malignancies (18, 19). Several studies have reported that lncRNAs are extensively expressed in tissues with high specificity and versatilely regulate gene expression, which plays a vital role in promoting or inhibiting tumor progression (20, 21). Xu et al. (22) indicated that lncRNAs with aberrant expression might facilitate cell proliferation, migration, or epithelial–mesenchymal transition (EMT) but suppress cell apoptosis and anticancer treatment sensitivity in PCa. So far, it has been universally reported that several lncRNAs such as CTBP1-AS, PCA3, GAS5, HOTAIR, and AR are linked to the occurrence and development of PCa (20, 22). However, the molecular function of lncRNAs remains unclear to date. Lately, increasing evidence has emerged to reveal the relationship between lncRNAs and genomic instability (23, 24). NORAD (25), a specific lncRNA activated by DNA damage, interacted with proteins concerning DNA repair and replication in steady-state cells and conduced to genomic stability by localizing to the nucleus on the condition of DNA damage. Another lncRNA, p53-responsive lncRNA (GUARDIN) (26), was reported to be essential for keeping genomic integrity upon exposure to exogenous genotoxicity. In addition, lncRNA DACOR1 was identified to contribute to aberrant DNA methylation, which could result in genomic instability (27). Moreover, convergence genomic instability and lncRNA SChLAP1 dysregulation underpinned the invasion of intraductal and cribriform PCa (28, 29). Although many lncRNAs have been documented to be associated with genomic instability, genomic instability-associated lncRNAs and their clinical outcome in cancers have not been elucidated thoroughly.

In this study, we intended to explore whether lncRNAs could indicate genomic instability and, thus, find their predictive value of BCR in PCa, based on somatic mutation data and lncRNA expression data extracted from The Cancer Genome Atlas (TCGA) database.

## Methods

### Data Acquisition

RNA-seq expression information, somatic mutation data, and clinical features with PCa were downloaded from the TCGA database (https://cancergenome.nih.gov/). We utilized the HTSeq-FPKM platform to retrieve the gene expression data of all the PCa with matched adjacent normal tissue samples. The masked somatic mutation information was acquired according to VarScan software and then visualized by the R package “maftools.” Tumor mutation burden (TMB) was defined as the number of somatic variants per million genome bases. We downloaded the common 318 transcription factors (TFs) from the Cistrome Cancer web resource (http://cistrome.org/CistromeCancer/) (30) and extracted its expression data from the RNA-seq expression profiles. The clinical characteristics included age, gender, T stage, N stage, Gleason score (GS), BCR survival status, and follow-up time.

### Identification of Differentially Expressed lncRNAs Associated With Genome Instability

We extracted lncRNAs from transcriptional data by uniquely matching them from the human genome. To confirm genomic instability-associated lncRNAs, we built a computational frame based on the mutator hypothesis combining somatic mutation data and lncRNA expression data in the PCa genome. Firstly, the cumulative somatic mutation number was calculated for patients with PCa, and the number of somatic mutations was the basis for ranking patients in descending order. Then the genomic unstable (GU) group was defined as the top 25% of patients, and the genomic stable (GS) group was defined as the last 25% of patients. The differentially expressed lncRNAs (|LogFC| > 1, false discovery rate (FDR) < 0.05) by comparing the GU group and the GS group were analyzed by using the “limma” R package. The “pheatmap” R package was utilized to depict the discrepancy of lncRNAs in the two groups. Ethics committee approval was not necessary as the data used in this study were downloaded from a public database (TCGA).

### Statistical Analysis

Hierarchical cluster analyses were conducted in samples with differentially expressed lncRNAs utilizing Ward’s linkage and Euclidean distance method. According to the median value of somatic mutation number, considered as the cutoff value, in the above samples, patients were separated into GU-like and GS-like groups. The “limma” and “ggpubr” R packages were utilized to present the difference in somatic mutation number and gene expression between the two groups.

All the PCa patients were equally divided into a train set and a test set. We performed univariate and multivariate Cox proportional hazard regression analyses to assess the correlation between the genomic instability-related lncRNA expression level and BCR. Here, a genomic instability-originated lncRNA signature (GILncSig), which referred to a prognostic risk score for PCa patients, was constructed to predict the outcome. The GILncSig of each sample was calculated as the summation of the product of prognostic lncRNA expression levels and coefficients (coefs). The coef was obtained from multivariate Cox regression and presented the lncRNA contribution to risk scores of BCR. We classified PCa patients into a high GILncSig (high-risk) group and a low GILncSig (low-risk) group according to the median value of GILncSig as a risk cutoff in the train set or test set.

The BCR survival of each prognostic risk group was estimated by the Kaplan–Meier (K-M) method with a log-rank test. The time-dependent receiver operating characteristic (ROC) curve was also utilized to evaluate the predictable performance of GILncSig. The univariate or log-rank test was used to assess the difference in somatic mutation number, specific gene expression, and TMB in the two risk groups. We also conducted multivariate Cox regression and stratified analysis to demonstrate the prognostic independence value of GILncSig from other critical clinical features. All the analyses were conducted with the statistical software package R.

### lncRNA–mRNA Correlations and Functional Enrichment Analysis

The co-expressed correlations between differentially expressed lncRNAs and mRNAs were computed by Pearson correlation coefficients, and the lncRNA-related partners were considered the top 10 mRNAs. We used the “igraph” R package to depict the co-expressed network of lncRNAs and mRNAs. To further investigate the biological functions of all correlated genes, functional enrichment analysis was performed to significantly determine the gene ontology (GO) and Kyoto Encyclopedia of Genes and Genomes (KEGG) pathway. GO analysis included biological process, cellular component, and molecular function. KEGG analysis referred to pathway enrichment. We utilized the clusterProfiler package in R software to perform the functional enrichment analysis.

### The lncRNA–Transcription Factor Regulatory Network

Pearson correlation analysis was conducted between the TFs and the lncRNAs associated with prognosis. A *P*-value less than 0.001 was considered to be significant. High-risk lncRNAs were defined as lncRNAs with a hazard ratio (HR) >1 and low-risk lncRNAs were defined as those with HR <1. A coefficient >0.4 was considered as positive regulation, and <0.4 was considered as negative regulation. The regulatory network of lncRNAs with prognostic value and related TFs was constructed and visualized utilizing the Cytoscape 3.9.1 software.

## Results

### Genomic Instability-Associated lncRNAs in PCa Patients

**Figure 1:**
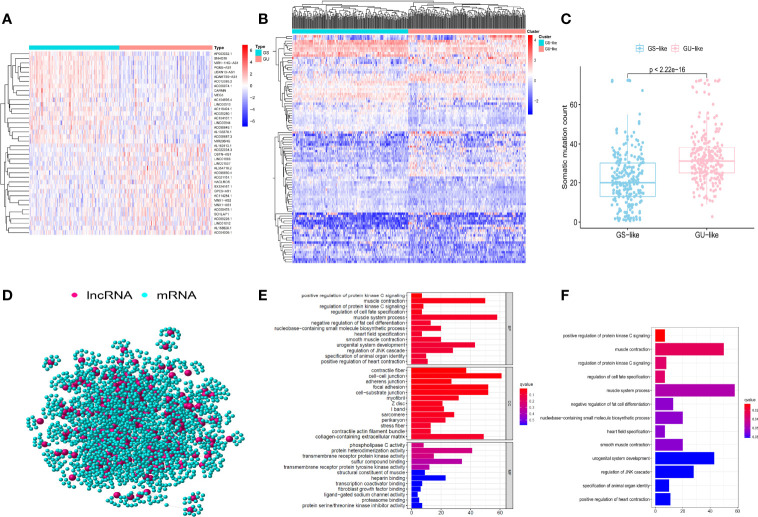
Identification and functional analysis of genomic instability-associated long non-coding RNAs (lncRNAs) in patients with prostate cancer (PCa). **(A)** 50 of the 95 significantly differentially expressed lncRNAs between the genomic unstable (GU) group and the genomic stable (GS) group. The left blue part is the GS-like group, and the right red part is the GU-like group. **(B)** Clustering of 488 PCa samples based on the expression profiles of 95 candidate genomic instability-associated lncRNAs. The left blue cluster is the GS-like group, and the right red cluster is the GU-like group. **(C)** Boxplots of somatic mutation count in the GU-like group and the GS-like group. **(D)** Co-expression network of genomic instability-associated lncRNAs and mRNAs. The red circles represent lncRNAs, and the blue circles represent mRNAs. GO **(E)** and KEGG **(F)** functional enrichment analyses for mRNAs co-expressing lncRNAs.

A total of 489 tumor tissue samples and 51 normal tissue samples were extracted from the TCGA database. According to the rank of the somatic mutation number in each patient, we assigned the top 25% (*n* = 126) and the last 25% (*n* = 122) samples into the GU group and the GS group. Then the lncRNA expression profiles were compared between these two groups to identify the lncRNAs with significant differences. A total of 95 significantly differentially expressed lncRNAs were confirmed by utilizing the SAM method. Of these, 34 lncRNAs were identified to be upregulated, while 61 lncRNAs were identified to be downregulated (**Supplementary Table 1**). **Figure 1A** shows part of the 95 significantly differentially expressed lncRNAs. Clustering analysis was performed on 489 PCa samples in the TCGA database utilizing the set of 95 lncRNAs with differential expression. Based on the expression levels of 95 differentially expressed lncRNAs, all 489 patients were clustered into two groups (**Figure 1B**). Then the somatic numbers of each sample were documented, whose median value was regarded as the cutoff point (**Supplementary Data 1**). Patients with higher somatic numbers were distributed into the GU-like group (*n* = 246), and the rest were distributed into the GS-like group (*n* = 243). As shown in **Figure 1C**, the GU-like group presented significantly higher somatic cumulative mutations than the GS-like group (median value: 30 vs. 20, *P* < 0.05).

To explore whether the competing endogenous regulating network exists in differentially expressed lncRNAs and mRNAs, we found the intersected lncRNAs and mRNAs and constructed the co-expression network (**Figure 1D**; **Supplementary Data 2**). lncRNAs and mRNAs were manifested as nodes, and they were linked together if they were related to each other. GO and KEGG analyses revealed the potential biological functions in the ceRNA network. GO analysis (**Figure 1E**) showed that the consensus genes were significantly associated with muscle system process, urogenital system development, regulation of JNK (c-Jun N-terminal kinase) cascade, and nucleobase-containing small-molecule biosynthetic process. In terms of KEGG pathway analysis, a significantly enriched pathway like regulation of protein kinase C (PKC) signaling was identified (**Figure 1F**). These pathways suggested that the altered expression of 95 significantly differentially expressed lncRNAs may influence the regulation of the cell cycle and play a critical role in gene damage and repair, which could result in an increase in genomic instability. Therefore, 95 differentially expressed lncRNAs could be regarded as candidate genomic instability-related lncRNAs (GIlncRNAs).

### A Signature Derived From Genomic Instability-Associated lncRNA for Prognosis

**Figure 2:**
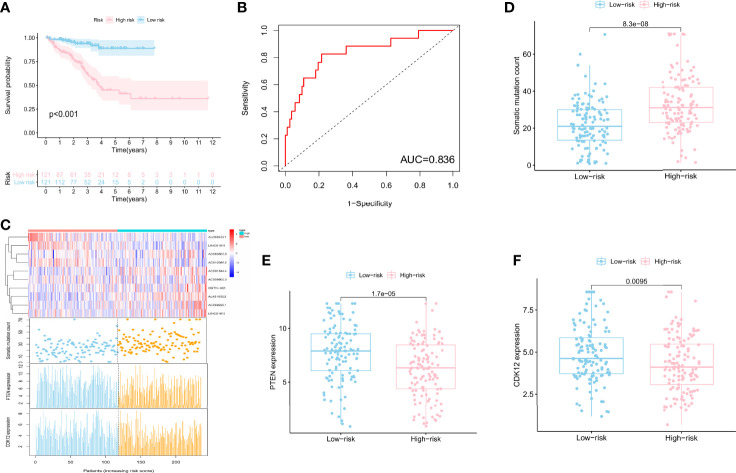
A signature derived from genomic instability-associated lncRNAs for biochemical recurrence in the train set. **(A)** Kaplan–Meier analysis of biochemical recurrence in patients with low or high risk predicted by the GILncSig in the train set. **(B)** ROC curve analysis of the GILncSig. **(C)** lncRNA expression patterns, the distribution of somatic mutation, and *PTEN* and *CDK12* expression with increasing GILncSig score. Somatic mutation count distribution **(D)**, *PTEN* expression **(E)**, and *CDK12* expression **(F)** in the high- and low-risk groups for PCa patients. The blue boxplot represents the high-risk group, and the red boxplot represents the low-risk group.

To find the outcome predictive value of these genomic instability-associated lncRNAs, 482 PCa patients with BCR survival status and time data from the TCGA database were assigned to the train set (*n* = 242) and the test set (*n* = 240) (**Supplementary Table 2**). Then we conducted univariate Cox proportional hazard regression analysis to investigate the relationship between genomic instability-related lncRNAs and BCR in the train set. Twenty-two genomic instability-related lncRNAs were significantly related to the BCR of PCa patients (*P* < 0.05; **Supplementary Figure 1**). Moreover, multivariate Cox proportional hazard regression analysis singled out 10 genomic instability-associated lncRNAs for further analysis (**Table 1**). We next developed a signature (GILncSig) derived from genomic instability-related lncRNAs and obtained the risk score of each patient in the train set. Utilizing the median risk score (1.347) as a threshold, the patients were classified into the high-risk group (*n* = 121) and the low-risk group (*n* = 121). K-M analysis revealed that patients in the low-risk group had a superior BCR survival than those in the high-risk group (*P* < 0.001; **Figure 2A**). The area under the curve (AUC) score was 0.836 in the ROC curve analysis of GILncSig (**Figure 2B**). Then we observed a change in the expression level of genomic instability-related lncRNAs, somatic mutation counts, and *PTEN* and *CDK12* expression with the GILncSig score increased (**Figure 2C**). Moreover, lncRNAs would present different expression patterns with the change in risk score. lncRNAs in red color represent an upregulated expression, while the blue color means the opposite. Comparison analysis in the two risk groups indicated significant discrepancies in somatic mutation count and *PTEN* and *CDK12* expression levels. Patients with high GILncSig score were prone to have higher somatic mutation number (30 vs. 22, *P* < 0.05) (**Figure 2D**) and lower expression levels of *PTEN* (*P* < 0.05) (**Figure 2E**) and *CDK12* (*P* < 0.05) (**Figure 2F**).

**Table 1:** Multivariate Cox regression analyses of the 10 of 95 genome instability-related lncRNAs correlated with biochemical recurrence in PCa.

Gene symbol	Coefficient	HR	95% CI	*P*-value
AL033523.1	−0.89	0.41	0.13–1.32	0.14
AC069228.1	0.08	1.08	1.04–1.12	**0.00**
LINC01018	−0.65	0.52	0.23–1.16	0.11
AC053503.3	0.14	1.15	1.07–1.23	**0.00**
AC091544.4	0.32	1.38	1.03–1.84	**0.03**
AL451050.2	0.70	2.01	1.33–3.02	**0.00**
AC009902.3	−0.69	0.50	0.25–1.00	**0.05**
AC012085.2	−0.52	0.60	0.29–1.22	0.16
LINC01612	0.10	1.11	1.01–1.21	**0.02**
OSTN-AS1	0.07	1.07	1.03–1.11	**0.00**

PCa, prostate cancer; GS, Gleason score; HR, hazard ratio; CI, confidence interval.

Bold values: P < 0.05. which means significant differences between groups.

**Figure 3:**
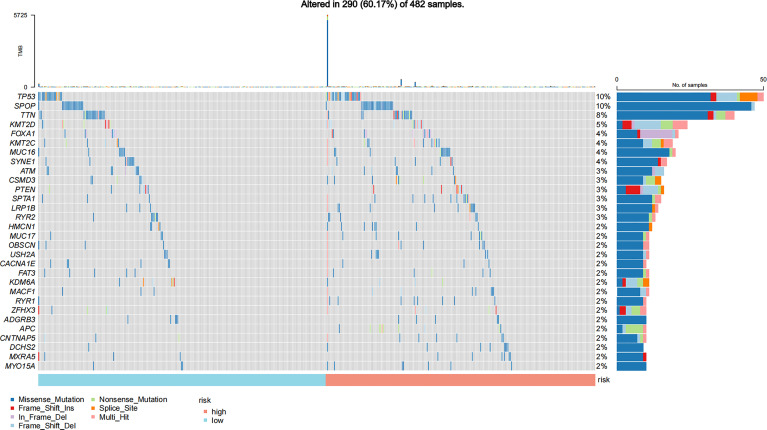
Mutation information of the top 30 genes in the high-risk and low-risk GILncSig groups is shown in the waterfall plot; various colors with annotations on the bottom represent the different mutation types. The bar plot exhibits the tumor mutation burden (TMB).

In addition, we also compared the mutation types (**Figure 3**), TMB (**Supplementary Figure 2**), and other hotspot gene mutations (**Supplementary Figure 3**) between the high- and low-risk GILncSig groups. The top 5 gene mutations were as follows: *TP53* (10%), *SPOP* (10%), *TTN* (8%), *KMT2D* (5%), and *FOXA1* (4%). A missense mutation was the most common variant. No significant gene mutation difference was found between these two groups (**Figure 3**). The high GILncSig risk group covered more distributions of mutation types (**Figure 3**) and had significantly higher TMB (**Supplementary Figure 2A**) and lower expression of *SPOP* (*P* < 0.05) (**Supplementary Figure 3A**) and *TMPRSS2* (*P* < 0.05) (**Supplementary Figure 3B**).

### Independent Verification of GILncSig in Two PCa Data Sets

**Figure 4:**
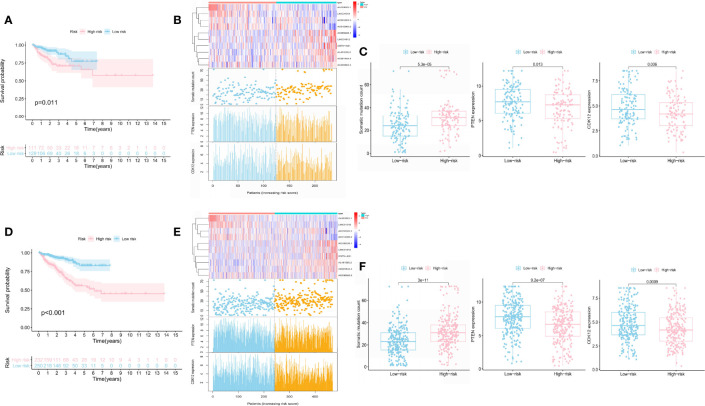
Prognostic performance of the GILncSig in the test set and the whole TCGA set. Kaplan–Meier analysis of biochemical recurrence in patients with low or high risk predicted by the GILncSig in the test set **(A)** and the TCGA set **(D)**. lncRNA expression profiles, somatic mutation count distribution, and *PTEN* and *CDK12* expression for patients in the high- and low-risk groups in the test set **(B)** and the TCGA set **(E)**. Somatic mutation distribution and *PTEN* and *CDK12* expression for patients in the high- and low-risk groups in the test set **(C)** and the TCGA set **(F)**.

We test the predictive value of GILncSig in the independent TCGA test set of 240 patients to validate its robustness. The test set applied the identical GILncSig and risk score cutoff that originated from the train set, of which 240 patients were divided into the high-risk group (*n* = 111) and the low-risk group (*n* = 129). Similar to the train set, patients with high GILncSig risk score suffered from inferior BCR survival than those with low GILncSig risk score (*P* = 0.011) (**Figure 4A**). The AUC score was 0.722 in the ROC curve analysis of the GILncSig (**Supplementary Figure 4A**). In the test samples, the GILncSig expression, somatic mutation count distribution, and *PTEN* and *CDK12* expression levels are presented in **Figure 4B**. TMB was also higher in the high-risk group (**Supplementary Figure 2B**). The somatic mutation count showed a significant difference in the high-risk group and the low-risk group (30 vs. 26, *P* < 0.05) (**Figure 4C**). *PTEN* (*P* = 0.013; **Figure 4C**), *CDK12* (*P* = 0.036; **Figure 4C**), *SPOP* (*P* < 0.05; **Supplementary Figure 3C**), and *TMPRSS2* (*P* < 0.05; **Supplementary Figure 3D**) expression levels were identified to be significantly lower in the high-risk group (**Figure 4C**).

The whole TCGA set showed a similar prognosis value of the GILncSig with the above results. The sample sizes of these two risk groups were 232 and 250, respectively. The BCR survival of patients with a higher risk score was shorter than those with a lower risk score (*P* < 0.001; **Figure 4D**). The AUC score was 0.771 in the ROC curve analysis of the GILncSig (**Supplementary Figure 4B**). In the test samples, the GILncSig expression, somatic mutation count distribution, and *PTEN* and *CDK12* expression levels are presented in **Figure 4E**. The high-risk group demonstrated significantly higher somatic mutation count (32 vs. 24, *P* < 0.05), higher TMB (**Supplementary Figure 2C**), and lower expression of *PTEN*, *CDK12*, *SPOP*, and *TMPRSS2* than the low-risk group (**Figure 4F** and **Supplementary Figures 3E, F**).

### Independent Verification of GILncSig in the Stratified Subgroups

**Figure 5:**
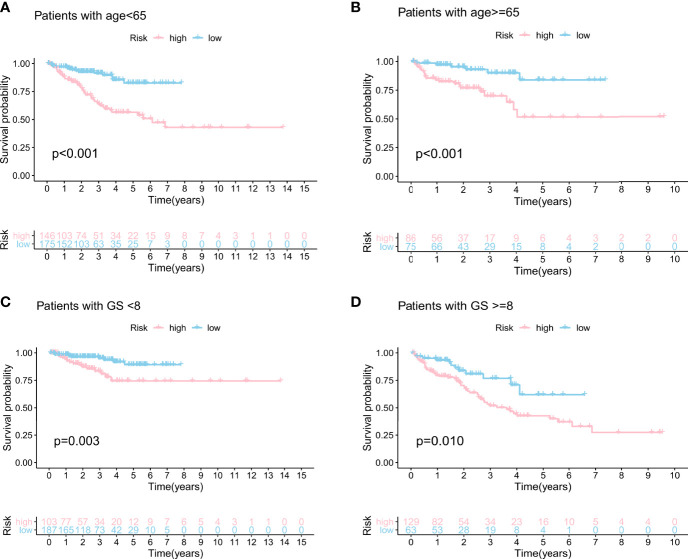
Stratification analyses by age and Gleason score. Kaplan–Meier curve analysis of biochemical recurrence in the high- and low-risk groups for patients <65 years of age **(A)** and patients ≥65 years of age **(B)**. Kaplan–Meier curve analysis of biochemical recurrence in the high- and low-risk groups for patients with GS <8 **(C)** and patients with GS ≥8 **(D)**.

We built the BCR survival model based on GILncSig and combined it with clinical features, including age, gender, T and N stages, and GS, by multivariate Cox regression analyses. The results showed that higher GILncSig was significantly related to poor BCR survival in the three sets (**Table 2**). However, the other two clinical factors, T stage and GS, were observed to be significantly associated with BCR in multivariate Cox regression analyses. In addition, we performed a stratification analysis to determine the independent prognostic value of GILncSig. Patients in the high-risk group were less likely to have favorable BCR survival compared to those in the low-risk group, regardless of age [<65 years, *n* = 321 (**Figure 5A**) vs. ≥65 years, *n* = 161 (**Figure 5B**)]. Next, patients were stratified by GS. In patients with GS less than 8 (*n* = 290) (**Figure 5C**), a higher GILncSig risk score was significantly correlated with unfavorable BCR survival. The same result was also verified in patients with GS more than 8 (*n* = 192) (**Figure 5D**).

**Table 2:** Univariate and multivariate Cox regression analyses of the GILncSig and biochemical recurrence in the whole TCGA set, train set, and test set.

Variables		Univariable model			Multivariable model		
		HR	95% CI	*P*-value	HR	95% CI	*P*-value
Whole TCGA set (*n* = 482)								
Age		1.01	0.98–1.04	0.57			
T stage	T3–T4/T1–T2	1.96	1.51–2.54	**0.00**	1.38	1.04–1.84	**0.03**
N stage	N1/N0	1.93	1.18–3.15	**0.01**	0.86	0.52–1.44	0.57
Gleason score	GS ≥ 8/GS < 8	4.77	3.01–7.57	**0.00**	3.66	2.14–6.26	**0.00**
GILncSig	High/low	4.64	2.80–7.72	**0.00**	3.86	2.19–6.81	**0.00**
Train set (*n* = 242)								
Age		1.00	0.96–1.04	0.94			
T stage	T3–T4/T1–T2	1.92	1.37–2.69	**0.00**	1.28	0.90–1.81	0.17
N stage	N1/N0	1.45	0.74–2.85	0.28			
GS	GS ≥ 8/GS < 8	7.12	3.56–14.23	**0.00**	4.65	2.24–9.66	**0.00**
GILncSig	High/low	5.24	2.55–10.77	**0.00**	3.17	1.51–6.65	**0.00**
Test set (*n* = 240)								
Age		1.02	0.97–1.08	0.34			
T stage	T3–T4/T1–T2	1.98	1.32–2.98	**0.00**	1.8	1.12–2.77	**0.01**
N stage	N1/N0	2.60	1.26–5.35	**0.01**	1.2	0.53–2.50	0.72
GS	GS ≥ 8/GS < 8	3.09	1.62–5.90	**0.00**	2.4	1.13–5.15	**0.02**
GILncSig	High/low	3.83	1.86–7.87	**0.00**	4.2	1.80–9.64	**0.00**

PCa, prostate cancer; GS, Gleason score; HR, hazard ratio; CI, confidence interval.

Bold values: P < 0.05. which means significant differences between groups.

### Comparison Between GILncSig and Previous lncRNA-Associated Features in Prognostic Performance

**Figure 6:**
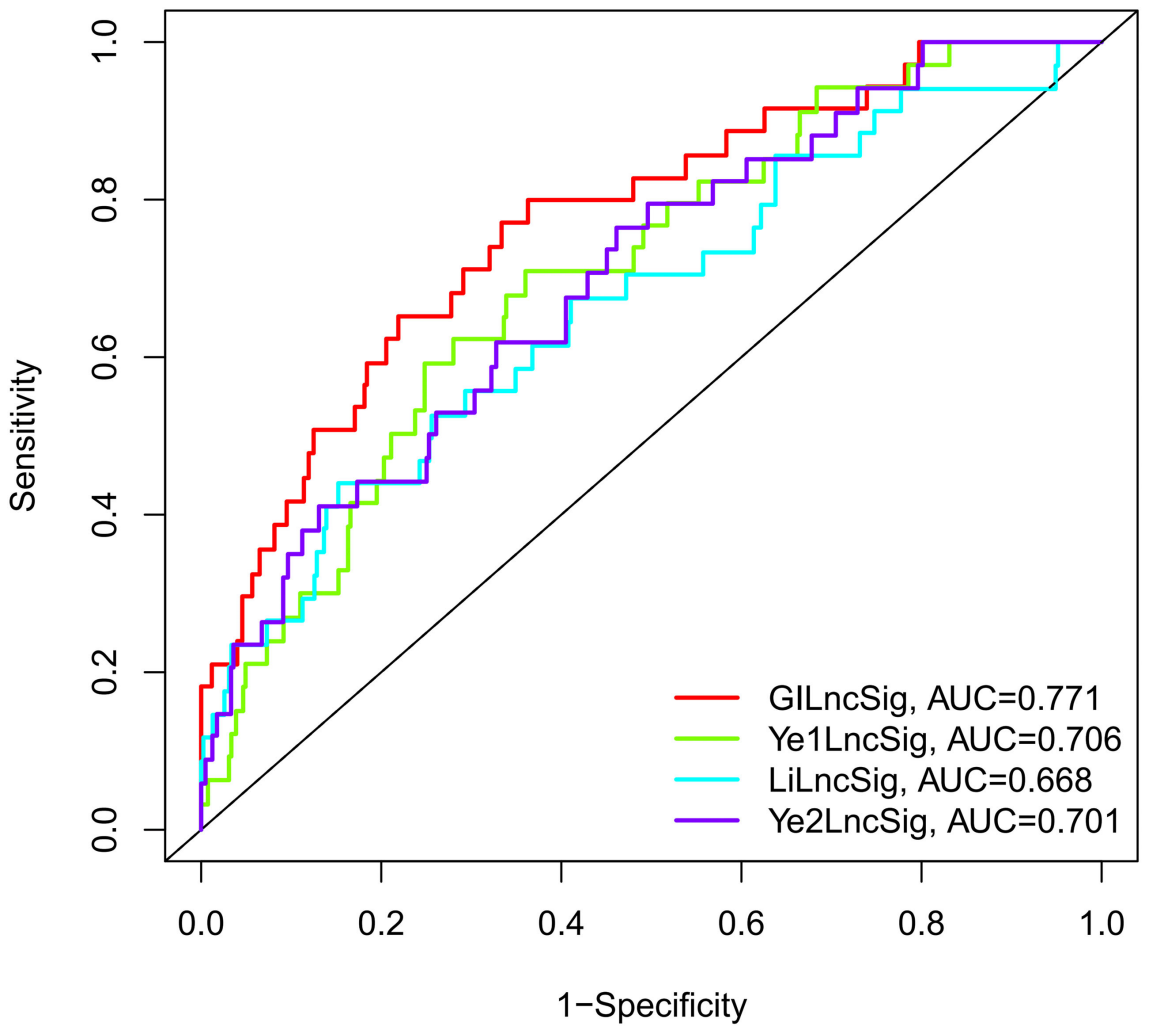
The ROC analysis at biochemical recurrence for the GILncSig, Ye1lncSig, Ye2lncSig, and LilncSig.

The prognostic performance of the GILncSig was further compared with three recently published lncRNA signatures: the 8-lncRNA signature extracted from Li’s study (31) (LilncSig), the 7-lncRNA signature extracted from Ye’s study (32) (Ye1lncSig), and the 5-lncRNA signature extracted from Ye’s study (33) (Ye2lncSig). **Figure 6** shows that the GILncSig AUC score of BCR was 0.771, which was significantly superior to that of Ye1lncSig (AUC = 0.706), Ye2lncSig (AUC = 0.701), and LilncSig (AUC = 0.668). The results indicated that the GILncSig in our study had better performance in predicting BCR than the three existing lncRNA signatures.

### The Prognostic Value of the GILncSig Survival Model Combined With *TP53* Mutation Status

**Figure 7:**
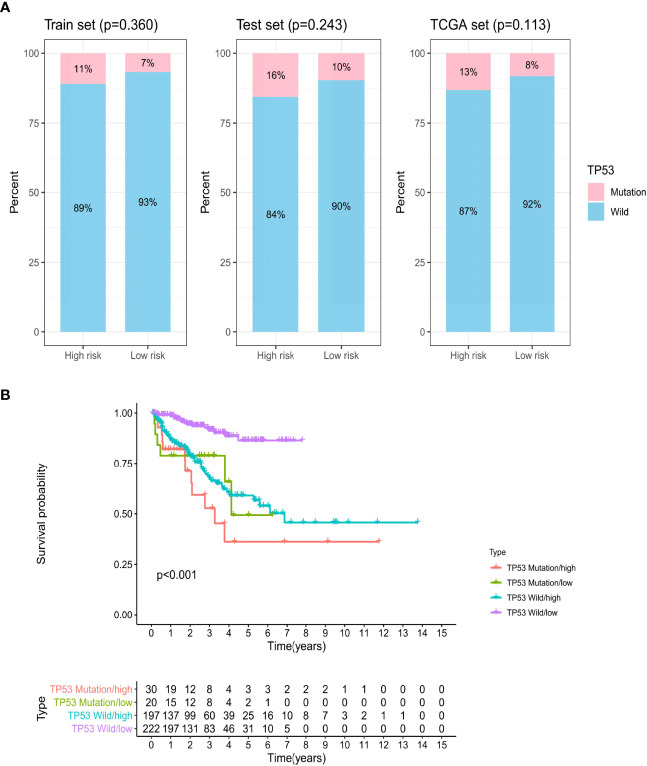
The biochemical recurrence model combined with GILncSig and *TP53* mutation status. **(A)** The proportion of *TP53* mutation in the high- and low-risk groups in the train set, test set, and TCGA set. **(B)** Kaplan–Meier curve analysis of biochemical recurrence is shown for patients stratified based on *TP53* mutation status and the GILncSig.

Further analysis showed that patients with *TP53* mutations constituted a larger proportion in the high-risk group than in the low-risk group among the three sets, although no significant statistical result was reached (**Figure 7A**). *TP53* was identified to be significantly associated with genomic instability and worse prognosis, which could be a potent predictor of PCa (34). Therefore, we combined *TP53* mutation status with the GILncSig BCR survival model to investigate the prognostic value. We divided patients into the *TP53*-sequence wild type (*TP53* wild) group and the *TP53*-sequence mutation type (*TP53* mutation) group. Then each *TP53* group was classified into the high-risk (high) group and the low-risk (low) group according to the GILncSig. **Figure 7B** presents the BCR survival curves of the four risk groups separated by *TP53* mutation status and GILncSig, consisting of the *TP53* wild/high group, *TP53* mutation/low group, *TP53* wild/low group, and *TP53* mutation/high group. In two risk groups, patients with *TP53* mutation suffer from an unfavorable BCR than patients with *TP53* wild. In the *TP53* mutation group and the *TP53* wild group, a high GILncSig score was significantly associated with poor BCR survival. Among these four groups, the *TP53* mutation/high group had the most inferior BCR survival, while the *TP53* wild/low group had the most superior BCR survival.

### Constructing a Regulatory Network of TFs and Prognostic lncRNAs

**Figure 8:**
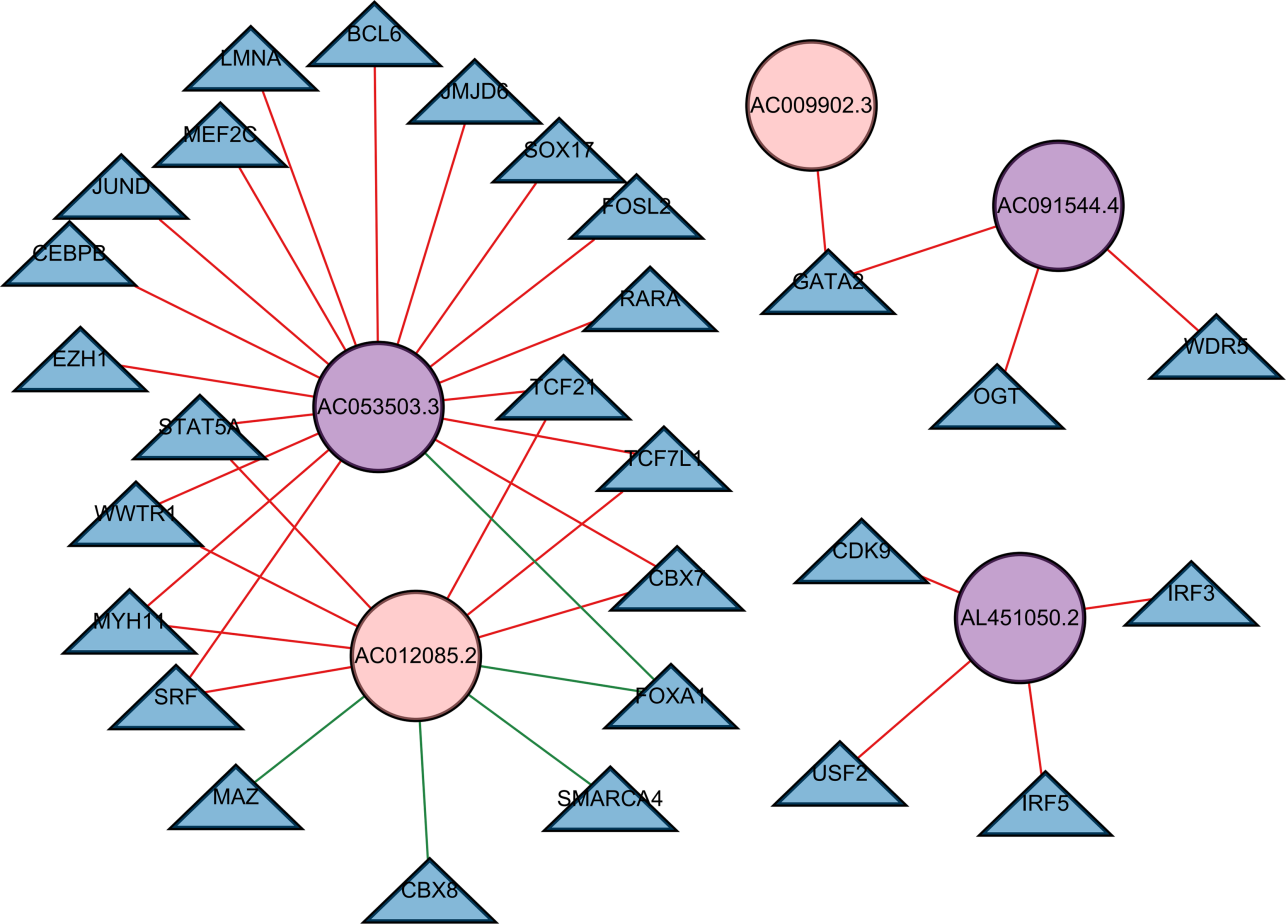
A transcription factor–lncRNA regulatory network. The regulatory network was constructed, which consisted of five lncRNAs with independent prognostic value. The blue nodes represent transcription factors, the purple nodes represent high-risk lncRNAs, and the pink nodes represent low-risk lncRNAs. Positive regulations between TFs and lncRNAs are displayed as red lines. Negative regulations are displayed as green lines.

We built a regulatory network of TFs and lncRNAs with independent prognostic value, involving 5 lncRNAs, 28 TFs, and 37 interactions (**Figure 8**; **Supplementary Data 3**). The regulatory network between all prognostic lncRNAs with TFs is shown in **Supplementary Figure 5**. The high-risk lncRNAs included AC053503.3, AL451050.2, and AC091544.4, while the low-risk lncRNAs included AC012085.2 and AC009902.3. A great number of TFs, covering CBX7, FOXA1, MYH11, SRF, STAT5A, TCF21, TCF7L1, and WWTR1, co-regulated AC053503.3 and AC012085.2. Among them, FOXA1 presented a negative regulation for these two lncRNAs. Moreover, CBX8, MAZ, and SMARCA4 negatively regulated the expression of AC012085.2.

## Discussion

Conventionally, clinical TNM stage, PSA at diagnosis, and GS were considered the most crucial prognostic features in PCa (35, 36), on which patients were classified into different risk groups and thereby received corresponding therapy. However, PCa is related to molecular diversity and considerable clinical heterogeneity (37–39). Different individuals with PCa could carry various genetic mutations that affect the progress of the tumor and the response to treatment (10). The phenomenon where DNA repair defects result in DNA mutation accumulation is called genomic instability (9). Several research studies have reported that genomic instability could be the ubiquitous characteristic of many cancers (40, 41) as well as the prognostic factor of PCa (38). Nevertheless, the reason for the generation of genomic instability and more genomic instability being presented by some types of PCa than others remains obscure. Furthermore, the quantitative evaluation of genomic instability degree has been a great challenge for researchers. Accumulating studies have indicated that aberrant epigenetic or transcriptional changes could cause genomic instability (25, 42, 43). Persistent efforts have been made to find the predicting signatures for genomic instability such as miRNAs and genomic instability-related protein-coding genes (PCGs) (10, 44).

Recently, lncRNAs have been identified as a vital part of tumor biology and associated with the occurrence and development of PCa (45, 46). The potential of lncRNAs in diagnosis and prognosis has been revealed in several studies (47, 48). With the growing understanding of lncRNAs in functional mechanisms, researchers gradually realized that lncRNAs were also of great importance for genomic instability, such as NORAD (25), GUARDIN (26), and SChLAP1 (28, 29). Although genomic instability-associated lncRNAs in genome-wide analysis and their clinical implication in PCa are still in infancy, some related studies have already been made.

A computing framework in our study, combining tumor mutation phenotype and lncRNA expression, was conducted to explore genomic instability-associated RNAs. Then we identified 95 novel genomic instability-related lncRNAs according to the profiles of lncRNA expression and somatic mutations of PCa. The functional and GO and KEGG analyses presented the enriched pathway of co-expressed genes with the 95 genomic instability-associated lncRNAs. We observed that these co-expressed genes were enriched in the regulation of the JNK cascade, nucleobase biosynthetic process, and regulation of PKC signaling and urogenital system development. The JNK pathway was clarified to be involved in DNA damage (49, 50). Ectopic JNK activation was found in genetically induced cervical intraepithelial neoplasias (CIN) (50). Furthermore, Kanchan et al. (51) also found an association between the JNK pathway and genomic instability. They indicated that JNK cascades were enhanced sequentially with the repetition of acquired copy number variations (CNVs) in the genomic regions. As for the PKC pathway, a previous finding (52) demonstrated that PKC was correlated to cell cycle activation and cell apoptosis induction. In particular, PKC could regulate transcription through the phosphorylation of various transcriptional factors containing the p53 tumor suppressor, which was essential for the arrest and apoptosis of the cell cycle in response to DNA damage. Moreover, PKC was reported to control DNA methylation patterns *via* cooperation with DNA methyltransferase 1 (DNMT1) (53). Therefore, the above enrichment pathways were all associated with genomic instability in PCa.

We further investigated the association between genomic instability-related lncRNAs and clinical outcome. An lncRNA signature (GILncSig) was constructed by 10 genomic instability-associated lncRNAs with prognostic value, stratifying patients into two risk groups in the train set with significantly different survival. The results were validated independently in the test set and the whole TCGA set. In addition, we found that the high GILncSig risk group had significantly higher TMB. Similarly, previous studies also established the lncRNA-related signature to explore the indicative functions of TMB (54–56). Ding et al. (54) and Yang et al. (55) calculated the multi-lncRNA classifier index and verified its predictive ability of TMB with accuracy (AUC = 0.70 and 0.99, respectively). The results showed that lower values of the classifier index were correlated to higher TMB, which presented favorable outcomes. However, in line with our results, another study (56) built an lncRNA prognostic signature and found that the higher lncRNA score risk group was associated with more gene mutations, which led to lower survival. Different modeling formulae might lead to discrepancies. As the current immunotherapy for PCa is still in its infancy, patients with inferior prognosis might obtain benefits from immunotherapy due to its high TMB with more potential therapeutic targets.

Specifically, we found that the GILncSig was significantly linked to the tumor mutation phenotype and to *PTEN*, *CDK12*, *SPOP*, and *TMPRSS2* expression in PCa, all of which are vital signs of genomic instability. Notably, patients in the high GILncSig risk group presented with low *PTEN*, *CDK12*, *SPOP*, and *TMPRSS2* expression.

*PTEN*, a multifunctional tumor suppressor, is identified to be the top frequent gene in PCa (57, 58). Nearly 70% of PCa patients were found to have a loss of expression of *PTEN* (59). Quite a few studies indicated that the loss of *PTEN* is significantly associated with higher GS (57), BCR (60), metastasis (61), and the development of neuroendocrine phenotype in PCa (62). Sun et al. (57) pointed out that *PTEN* is usually mutated by copy number loss instead of point mutation. They investigated the expression level of *PTEN* mRNA in the *PTEN* wild and mutation groups. The results revealed that the downregulated mRNA expression level occurred in the mutation group. In addition, Jia et al. (63) found the upregulated lncRNA MCM3AP-AS1 in PCa tissue, which could inhibit *PTEN* expression. Therefore, patients in the high GILncSig risk group could reveal low *PTEN* expression in the three sets.

*CDK12* is seldom altered across solid cancers but occurs in 4%–11% of PCa patients and is more common in metastatic castration-resistant PCa (mCRPC) (64, 65). Nguyen et al. (65) performed a pan-cancer analysis of *CDK12* mutation and found that CDK-mutated PCa was significantly related to more aggressive clinical characteristics and poor overall survival (OS). *CDK12* encodes a cyclin-dependent serine/threonine kinase that participates in DNA repair regulation by homologous recombination (HR) (66). Increasing evidence suggested that the loss of biallelic *CDK12* (*CDK12*-bi), characterized by high genomic instability and tandem duplications, could determine a specific phenotype in PCa with high response to immune checkpoint inhibitors due to the high neoantigen burden (37, 64). Notably, *CDK12*-bi was significantly related to a higher breakpoint number and smaller copy number-altered segment size (65). The above evidence may explain why low CDK expression existed in the high GILncSig risk group in our results. Moreover, inactivating missense mutations in *SPOP* is one of the most common gene mutations in PCa, especially in the localized PCa (67). *SPOP* inactivation leads to overexpression of the androgen receptor (AR) and further promotes cell proliferation (68). Furthermore, *SPOP* was reported to be involved in DNA double‐strand break repair, implying that mtSPOP PCa featured genomic instability and might be sensitive to PARP inhibitors and other synthetic lethal therapies (10). *TMPRSS2* is one of the androgen regulatory genes, participating in tumor invasion and metastasis. The infusion of *TMPRSS2* and *ERG* (*TMPRSS2*–*ERG*) was considered the critical driver of prostate oncogenesis, occurring in about half of PCa cases (69). It was also associated with genomic instability, which led to a more advanced tumor stage and less disease survival (70).

Among the 10 genomic instability-associated lncRNAs with prognostic value, only LINC01018, OSTN-AS1, and AC012085.2 were reported in previous research (71, 72), and the others were novel lncRNAs that were related to genomic instability and could predict the outcome of PCa. LINC01018 was found to have the ability to regulate critical genes in gastric cancer (73) and hepatocellular carcinoma (72). OSTN-AS1 was identified to conduct the biological process of sequence-specific DNA binding in PCa (71). AC012085.2, one of the autophagy lncRNAs, was found to be upregulated in PCa compared to benign prostatic hyperplasia (BPH) tissues (74). These literature-mining results, along with the above validation results in various data sets, demonstrated that the GILncSig could predict the BCR and also had the potential to be a genomic instability indicator for PCa patients.

In our study, we also constructed a GILncSig survival model combined with *TP53* mutation status. Patients with *TP53* mutation would have significantly shorter BCR survival than those with *TP53* wild type both in the high-risk and low-risk groups. These results were in line with previous evidence. *TP53* mutations have been identified to confer poor response for CRPC in patients who received AR-targeted therapy (75). A high GILncSig risk score was significantly correlated with unfavorable outcome in both the *TP53* mutation and *TP53* wild groups. Therefore, patients in the *TP53* mutation/high group showed the most inferior BCR survival since the most significant factors associated with unfavorable prognosis were concentrated on them, and patients in the *TP53* wild/low group merited the best BCR survival.

TFs exert vital effects in regulating the expression of lncRNAs *via* specific binding sites in lncRNA transcripts, which reasonably participated in tumor development. The correlation between them is still being explored. As for PCa, a TF named HOXB13 has been found to regulate the lncRNA HOXA11-AS for promoting bone metastasis in PCa, by CCL2/CCR2 cytokine regulation and integrin signaling in autocrine and paracrine processes (76). In recent years, a TF-based regulatory network has been established to clarify the underlying regulation circuits and mechanisms (77–80). Ning et al. (81) constructed a feedforward network mediated by lncRNA and illustrated that many TF–lncRNA interactions were implicated in prognostic motifs. Jiang et al. (77) identified a prognostic signature containing 16 TFs and constructed an interplay network with lncRNAs in PCa. 839 lncRNAs and 124 TFs with 11,398 interactions were found in primary PCa. MYC and its lncRNA partner AL590617.2 were validated by RNA immunoprecipitation quantitative PCR (RIP-qPCR) in LNCaP PCa cells. In our study, we found some TFs related to independent prognostic lncRNAs, in which the prognostic value of FOXA1, JUND, and SRF had been clarified by Jiang et al. Nonetheless, given the complexity of the transcriptional control, the interplay should be contemplated cautiously.

Although we provided critical insights to assess genomic instability and the BCR of PCa patients, some limitations still exist that need to be considered. Firstly, we only validated the GILncSig in the TCGA database. More independent databases with abundant data of lncRNAs are required to be involved to ensure the robustness and reproducibility of the data in the future. Secondly, the GILncSig was constructed on the basis of the mutator hypothesis utilizing the computational frame; thus, more functional studies are warranted to be conducted by experimental biologists, contributing to figuring out the regulation mechanism of the GILncSig in keeping genomic stability.

## Conclusions

In conclusion, this study identified genomic instability-related lncRNAs based on a mutator hypothesis-induced calculative approach, providing a vital method for further studies evaluating the impact of lncRNAs on genomic instability. We combined the lncRNA expression data with somatic mutation information and clinical features of PCa. Next, we constructed a signature (GILncSig) derived from genomic instability-associated lncRNAs as a BCR predictor to classify risk groups for PCa patients. The results were verified on different independent sets successfully. The GILncSig may exert profound effects on genomic instability and personalized management in PCa patients through further prospective validation.

## Data Availability Statement

All the data of this study can be downloaded from TCGA (https://portal.gdc.cancer.gov/) and the Cistrome Cancer web resource (http://cistrome.org/).

## Author Contributions

LY and JL were responsible for the study design and protocol. WL, HX, XZ, and LT were responsible for the data collection, analysis, and interpretation. LT, WL, HX, XZ, SQ, WH, QW, and JA participated in manuscript draft preparation and revision. LY and JL funded this project. All authors contributed to the article and approved the submitted version.

## Funding

This work was supported by the National Key Research and Development Program of China (Grant No. 2017YFC0908003); National Natural Science Foundation of China (Grant Nos. 81902578, 81974098, 8197032158); China Postdoctoral Science Foundation (2017M612971); Post-Doctoral Science Research Foundation of Sichuan University (2020SCU12041); Post-Doctoral Research Project, West China Hospital, Sichuan University (2018HXBH084); and National Clinical Research Center for Geriatrics, West China Hospital, Sichuan University (Z2018C01).

## Conflict of Interest

The authors declare that the research was conducted in the absence of any commercial or financial relationships that could be construed as a potential conflict of interest.

## Publisher’s Note

All claims expressed in this article are solely those of the authors and do not necessarily represent those of their affiliated organizations, or those of the publisher, the editors and the reviewers. Any product that may be evaluated in this article, or claim that may be made by its manufacturer, is not guaranteed or endorsed by the publisher.

## Supplementary Material

Supplementary Figure 1 Forest plot describing the association between lncRNAs and biochemical recurrence.

Supplementary Figure 2 Tumor mutation burden (TMB) in two GILncSig groups. **(A)** The train set. **(B)** The test set **(C)** The TCGA set.

Supplementary Figure 3 SPOP and TMPRSS2 expression in two GILncSig groups. **(A, B)** The train set. **(C, D)** The test set. **(E, F)**The TCGA set.

Supplementary Figure 4
**(A)** ROC curves analysis of the GILncSig in the test set. **(B)** ROC curves analysis of the GILncSig in the whole TCGA set.

Supplementary Figure 5 A transcription factors-lncRNAs regulatory network. Constructed a regulatory network of lncRNAs with prognostic value. Blue nodes represent transcription factors, purple nodes represent high-risk lncRNAs, pink nodes represent low-risk lncRNAs. Positive regulations between TFs and lncRNAs are displayed as red lines. Negative regulations are displayed as green lines.

Supplementary Data Sheet 2 LncRNAs-mRNAs network.

Supplementary Data Sheet 3 TFs-lncRNAs network.

## Abbreviations

lncRNAs, long non-coding RNAs; GILncSig, genomic instability-derived lncRNA signature; PCa, prostate cancer; BCR, biochemical recurrence; GI, genomic instability; CIN, chromosome instability; CIN, cervical intraepithelial neoplasias; EMT, epithelial–mesenchymal transition; TCGA, The Cancer Genome Atlas; GS, Gleason score; GS, genomic stable; GU, genomic unstable; OS, overall survival; coefs, coefficients; ROC, receiver operating characteristic; JNK, c-Jun N-terminal kinase; PKC, protein kinase C; GIlncRNAs, GI-associated lncRNAs; AUC, area under the curve; PCGs, protein-coding genes; CNVs, copy number variations; DNMT1, DNA methyltransferase 1; mCRPC, metastatic castration-resistant PCa; HR, homologous recombination; *CDK12*-bi, biallelic *CDK12*; AR, androgen receptor; TMB, tumor mutation burden; TFs, transcription factors
