# Supplementary figures and images for: Mutator-Derived lncRNA Landscape: A Novel Insight Into the Genomic Instability of Prostate Cancer
Source: Front Oncol. 2022 Jul 4;12:876531. doi: 10.3389/fonc.2022.876531 (PMC9291324; doi:10.3389/fonc.2022.876531)

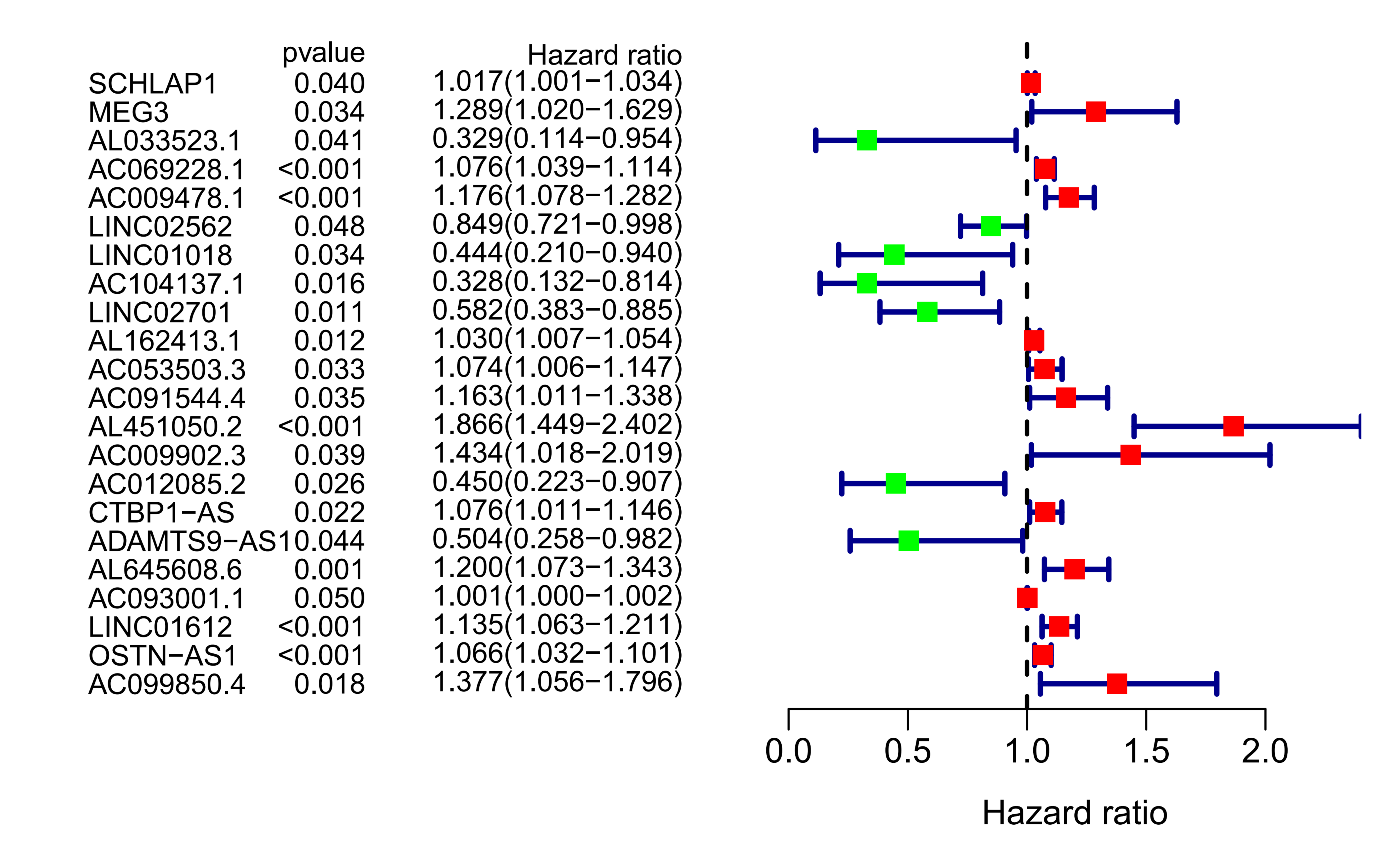

Supplement: Supplementary Figure 1 — Forest plot describing the association between lncRNAs and biochemical recurrence. [file Image_1.tiff]

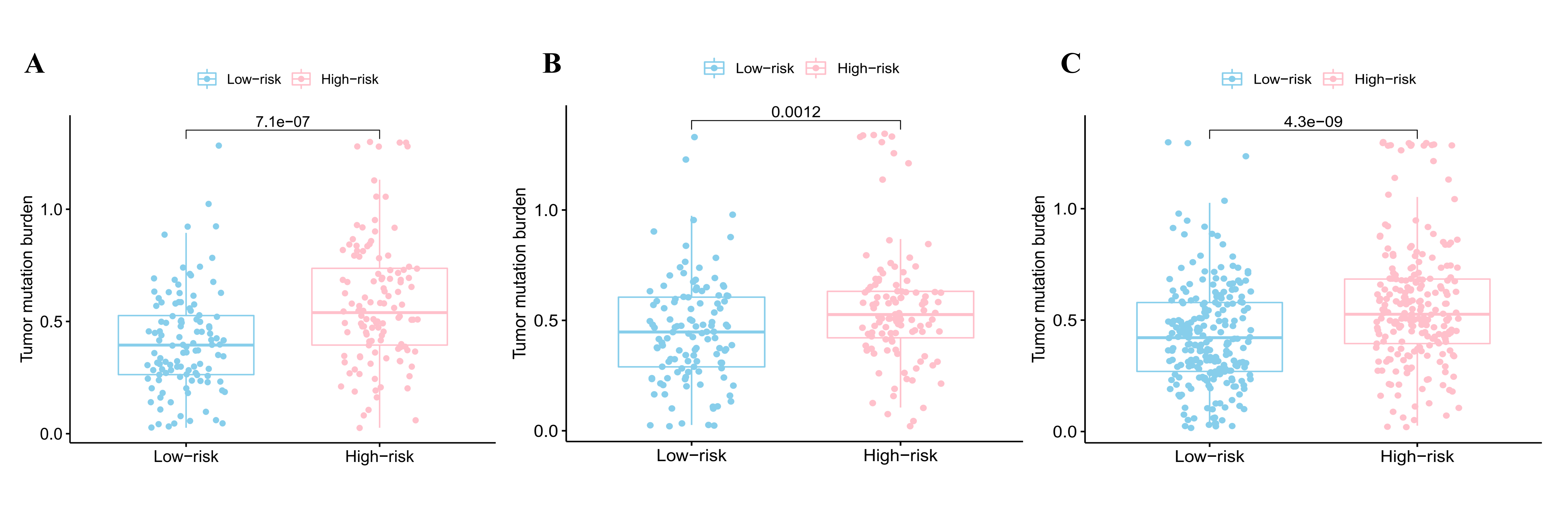

Supplement: Supplementary Figure 2 — Tumor mutation burden (TMB) in two GILncSig groups. (A) The train set. (B) The test set (C) The TCGA set. [file Image_2.tiff]

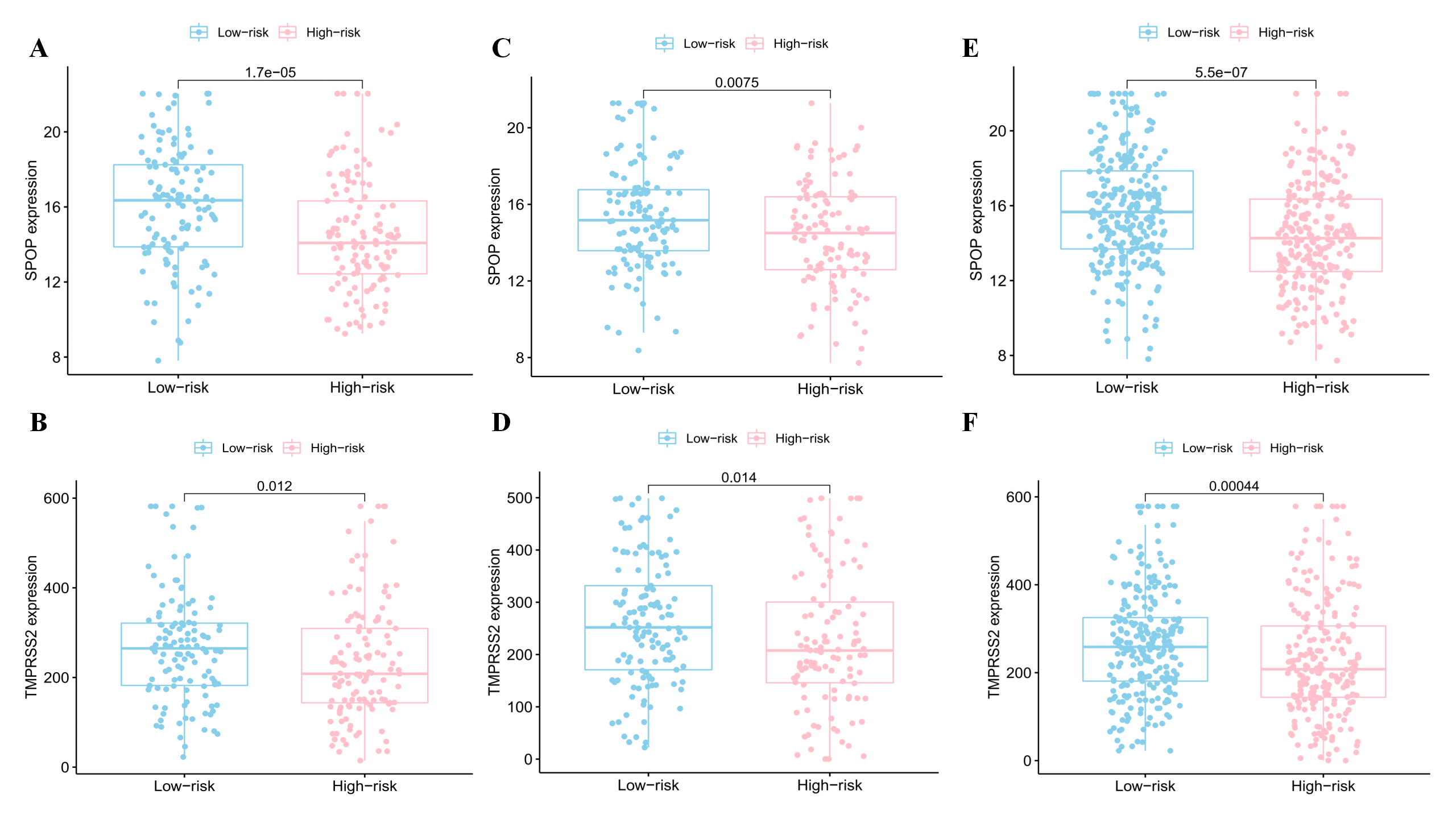

Supplement: Supplementary Figure 3 — SPOP and TMPRSS2 expression in two GILncSig groups. (A, B) The train set. (C, D) The test set. (E, F)The TCGA set. [file Image_3.tiff]

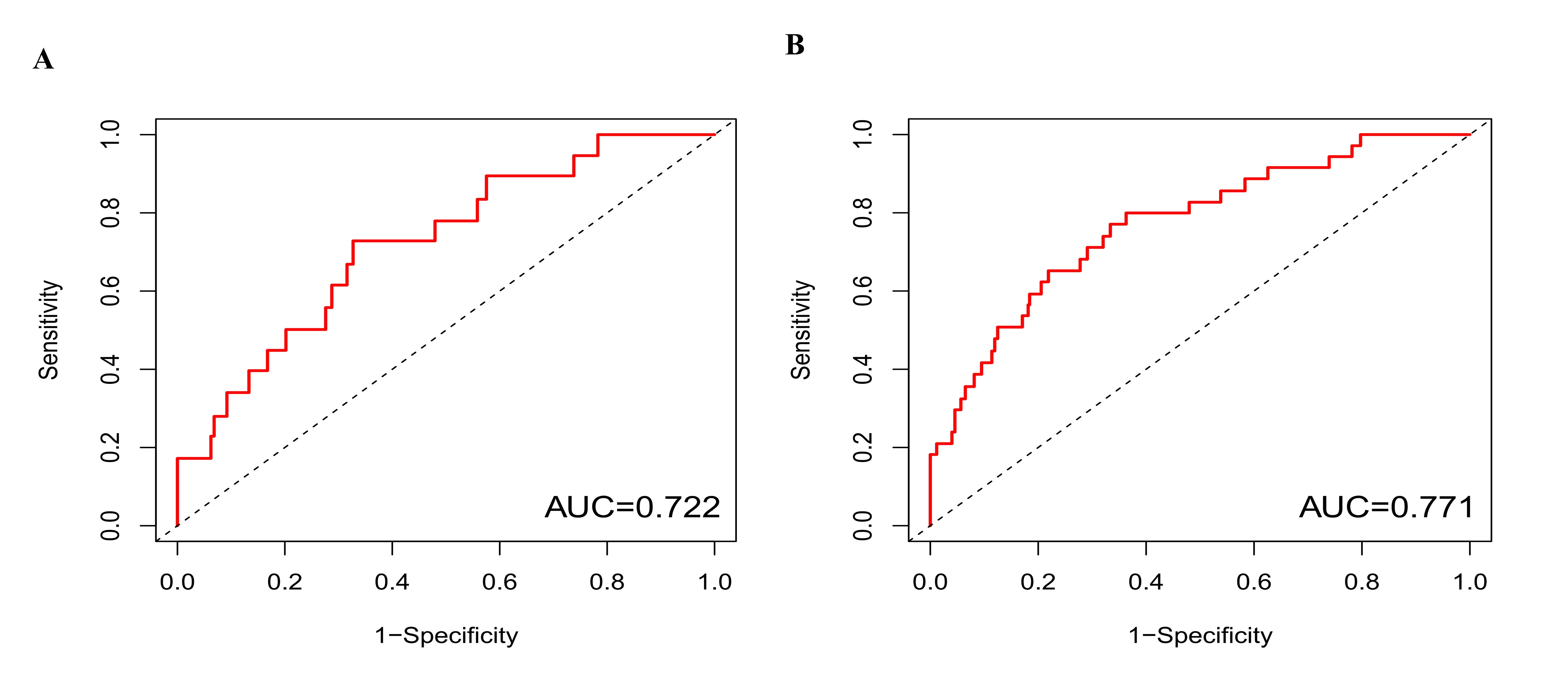

Supplement: Supplementary Figure 4 — (A) ROC curves analysis of the GILncSig in the test set. (B) ROC curves analysis of the GILncSig in the whole TCGA set. [file Image_4.tiff]

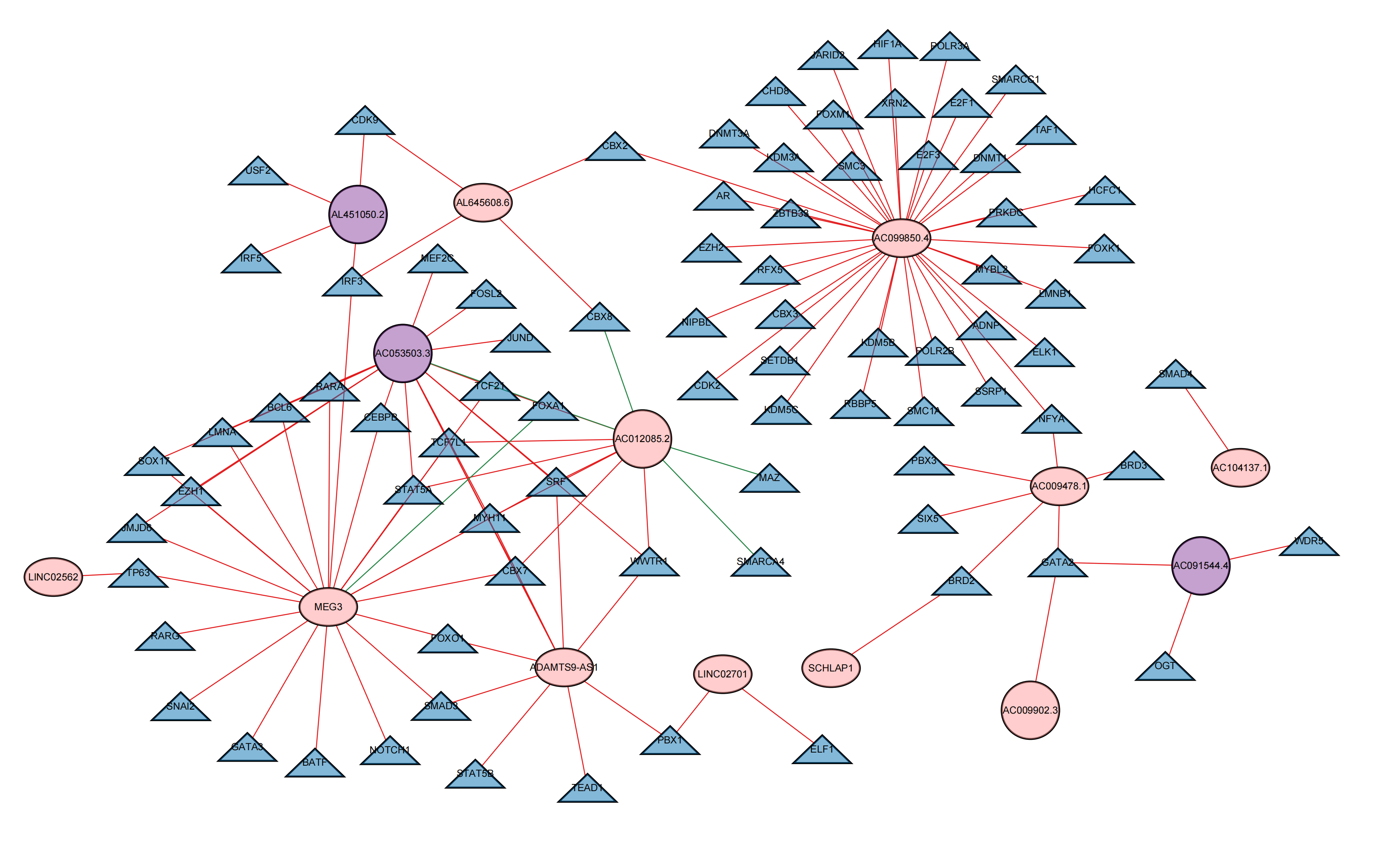

Supplement: Supplementary Figure 5 — A transcription factors-lncRNAs regulatory network. Constructed a regulatory network of lncRNAs with prognostic value. Blue nodes represent transcription factors, purple nodes represent high-risk lncRNAs, pink nodes represent low-risk lncRNAs. Positive regulations between TFs and lncRNAs are displayed as red lines. Negative regulations are displayed as green lines. [file Image_5.tiff]
