# Supplementary material for: Mutator-Derived lncRNA Landscape: A Novel Insight Into the Genomic Instability of Prostate Cancer
Source: Front Oncol. 2022 Jul 4;12:876531. doi: 10.3389/fonc.2022.876531 (PMC9291324; doi:10.3389/fonc.2022.876531)
Supplement: Supplementary file 9 [file Table_1.docx]

**Supplement Table 1.** 95 differentially expressed lncRNAs between genomic unstable group and genomic stable group

| **Lnc** | **conMean** | **treatMean** | **logFC** | **pValue** | **fdr** | **Regulate** |
| --- | --- | --- | --- | --- | --- | --- |
| SCHLAP1 | 1.78665510 | 8.97455375 | 2.32857904 | 0.00048237 | 0.0020692 | Up |
| AC080013.1 | 0.68856824 | 0.29869005 | -1.20495045 | 0.00000000 | 0.00000000 | Down |
| AC008687.3 | 0.68147417 | 0.20408389 | -1.73949666 | 0.00000000 | 0.00000001 | Down |
| PGM5-AS1 | 19.27761187 | 4.92272846 | -1.96939627 | 0.00000000 | 0.00000000 | Down |
| AC015845.2 | 1.80097132 | 0.83287289 | -1.11260697 | 0.00000000 | 0.00000000 | Down |
| MEG3 | 2.26545741 | 0.78845660 | -1.52269911 | 0.00000000 | 0.00000002 | Down |
| AC021151.1 | 0.29830736 | 0.78318105 | 1.39254629 | 0.00257029 | 0.00796846 | Up |
| LINC00513 | 0.69700265 | 0.22825213 | -1.61053585 | 0.00007885 | 0.00044609 | Down |
| AL033523.1 | 1.05882383 | 0.49471897 | -1.09778146 | 0.00005098 | 0.00030682 | Down |
| AC069228.1 | 0.74883885 | 2.23252055 | 1.57594627 | 0.00807775 | 0.02057794 | Up |
| LINC00844 | 16.90342094 | 6.18957322 | -1.44940341 | 0.00000000 | 0.00000000 | Down |
| GPC5-AS1 | 1.80734637 | 12.58638820 | 2.79991942 | 0.00001327 | 0.00009885 | Up |
| AL132822.1 | 0.58799949 | 0.24525923 | -1.26150744 | 0.00000011 | 0.00000162 | Down |
| AC005180.2 | 9.05563545 | 4.02026111 | -1.17152667 | 0.00000000 | 0.00000000 | Down |
| FENDRR | 2.87716715 | 1.32128884 | -1.12270316 | 0.00000000 | 0.00000000 | Down |
| AC009478.1 | 0.60916688 | 2.11455177 | 1.79544247 | 0.00001981 | 0.00013609 | Up |
| AC098649.1 | 2.08535487 | 0.41358773 | -2.33402764 | 0.00197261 | 0.00637355 | Down |
| UBXN10-AS1 | 3.61419625 | 0.95426068 | -1.92121951 | 0.00000000 | 0.00000000 | Down |
| AC114284.1 | 0.60777294 | 1.52659615 | 1.32871411 | 0.00000226 | 0.00002233 | Up |
| AC104695.4 | 3.67557984 | 1.27924084 | -1.52268396 | 0.00454700 | 0.01271853 | Down |
| LINC02562 | 3.38438548 | 1.48827018 | -1.18525745 | 0.00000000 | 0.00000000 | Down |
| AC107464.2 | 1.02907864 | 0.46664082 | -1.14096882 | 0.00000000 | 0.00000000 | Down |
| AP003032.1 | 0.59260114 | 0.21760084 | -1.44537729 | 0.00000000 | 0.00000000 | Down |
| AC135803.1 | 0.97740730 | 0.48251792 | -1.01837736 | 0.00000002 | 0.00000036 | Down |
| LINC01018 | 0.83658747 | 0.40088573 | -1.06132532 | 0.00000000 | 0.00000000 | Down |
| AL158828.1 | 0.31208809 | 0.94028909 | 1.59115106 | 0.00003230 | 0.00020868 | Up |
| AC104137.1 | 0.63141293 | 0.22996739 | -1.45715449 | 0.00000000 | 0.00000000 | Down |
| AF001548.1 | 1.44406050 | 0.66818255 | -1.11181696 | 0.00000000 | 0.00000002 | Down |
| AC087392.1 | 0.63613161 | 0.31299881 | -1.02316808 | 0.00000000 | 0.00000005 | Down |
| MPPED2-AS1 | 1.40733042 | 0.57860220 | -1.28231737 | 0.00000000 | 0.00000000 | Down |
| AC020916.1 | 10.12800162 | 4.89121221 | -1.05008558 | 0.00161828 | 0.00540539 | Down |
| AL365181.4 | 0.99813958 | 0.39660243 | -1.33154807 | 0.00000000 | 0.00000000 | Down |
| AC036108.3 | 1.15823392 | 0.51170223 | -1.17855024 | 0.00000000 | 0.00000000 | Down |
| MIR205HG | 13.72363101 | 4.32355433 | -1.66637252 | 0.00000000 | 0.00000000 | Down |
| LSAMP-AS1 | 1.50519257 | 0.71085291 | -1.08232510 | 0.00000000 | 0.00000000 | Down |
| AP001107.5 | 1.78358043 | 0.73312554 | -1.28264411 | 0.00000000 | 0.00000000 | Down |
| LINC01687 | 0.26953404 | 0.57114385 | 1.08338665 | 0.00784233 | 0.02005986 | Up |
| AL033519.4 | 0.64935803 | 0.29638171 | -1.13155773 | 0.00000092 | 0.00001071 | Down |
| BX324167.1 | 0.36515740 | 1.04043419 | 1.51059535 | 0.00024035 | 0.00112860 | Up |
| MIR1-1HG-AS1 | 1.09230909 | 0.41138459 | -1.40882150 | 0.00000000 | 0.00000000 | Down |
| MNX1-AS1 | 0.92446790 | 2.47227832 | 1.41914604 | 0.00000000 | 0.00000002 | Up |
| AC078993.1 | 1.05272690 | 2.58083283 | 1.29370548 | 0.00006907 | 0.00039842 | Up |
| LINC02701 | 1.40074296 | 0.67615775 | -1.05076047 | 0.00000003 | 0.00000044 | Down |
| WNT5A-AS1 | 0.82986739 | 1.68346246 | 1.02047884 | 0.00111176 | 0.00393672 | Up |
| AL162413.1 | 1.48757124 | 3.94259568 | 1.40618700 | 0.00004007 | 0.00025045 | Up |
| KRT7-AS | 1.11731962 | 0.48109322 | -1.21565356 | 0.00101841 | 0.00365278 | Down |
| EPHA5-AS1 | 0.97338016 | 0.37357462 | -1.38160692 | 0.00000000 | 0.00000000 | Down |
| AC053503.3 | 4.86391640 | 2.39208949 | -1.02384707 | 0.00000002 | 0.00000034 | Down |
| AC119424.1 | 0.80659944 | 0.07617168 | -3.40452575 | 0.00000000 | 0.00000002 | Down |
| Z83839.2 | 1.47833536 | 0.61905832 | -1.25582635 | 0.00000001 | 0.00000021 | Down |
| AC005180.1 | 5.81968456 | 2.67717170 | -1.12023129 | 0.00000000 | 0.00000000 | Down |
| AC022034.3 | 0.56040519 | 5.50290378 | 3.29565089 | 0.00008066 | 0.00045215 | Up |
| AC090527.4 | 2.07219329 | 0.91363316 | -1.18147167 | 0.00059448 | 0.00237304 | Down |
| AC009974.1 | 0.65023560 | 0.21617937 | -1.58873366 | 0.00000000 | 0.00000000 | Down |
| AC007750.1 | 0.70376170 | 0.28811843 | -1.28842503 | 0.00000046 | 0.00000570 | Down |
| AC091544.4 | 0.69817088 | 1.44983530 | 1.05423694 | 0.00057160 | 0.00229585 | Up |
| AL451050.2 | 0.29923021 | 0.61259390 | 1.03367517 | 0.00001895 | 0.00013109 | Up |
| AC104117.3 | 0.80710137 | 0.34315148 | -1.23390432 | 0.00000000 | 0.00000000 | Down |
| AL445426.1 | 0.63795342 | 0.31757410 | -1.00635782 | 0.00000000 | 0.00000001 | Down |
| HAGLROS | 0.43908082 | 1.17162068 | 1.41594714 | 0.00003437 | 0.00021977 | Up |
| LINC01507 | 0.28166508 | 0.90389806 | 1.68217939 | 0.00023753 | 0.00111989 | Up |
| AL354710.2 | 0.03187857 | 1.09813187 | 5.10632059 | 0.00127105 | 0.00442027 | Up |
| AC004009.1 | 0.28701745 | 0.87751789 | 1.61229007 | 0.00097243 | 0.00352031 | Up |
| PLBD1-AS1 | 0.72284885 | 0.30761102 | -1.23258683 | 0.00000000 | 0.00000003 | Down |
| AL049555.1 | 1.19143088 | 0.48687315 | -1.29107740 | 0.00000000 | 0.00000000 | Down |
| AC009902.3 | 0.25858857 | 0.56414754 | 1.12541400 | 0.00001109 | 0.00008524 | Up |
| AC109361.2 | 0.46161109 | 1.01896630 | 1.14235653 | 0.00031753 | 0.00143553 | Up |
| PCAT7 | 1.49755786 | 3.14589798 | 1.07086014 | 0.00000000 | 0.00000000 | Up |
| FGF14-AS2 | 1.76943732 | 0.83057014 | -1.09111674 | 0.00000000 | 0.00000000 | Down |
| AL354766.2 | 2.39927816 | 1.12685549 | -1.09029791 | 0.00000000 | 0.00000000 | Down |
| ACTA2-AS1 | 2.35616551 | 1.05642848 | -1.15724578 | 0.00000000 | 0.00000003 | Down |
| AC244517.1 | 0.30504093 | 0.64998238 | 1.09139778 | 0.00005643 | 0.00033341 | Up |
| AC012085.2 | 1.12891079 | 0.43260715 | -1.38380207 | 0.00000000 | 0.00000000 | Down |
| MNX1-AS2 | 0.99666310 | 2.55361400 | 1.35736265 | 0.00000007 | 0.00000115 | Up |
| AC025280.1 | 1.56494409 | 0.22889996 | -2.77332202 | 0.00000000 | 0.00000002 | Down |
| CTBP1-AS | 1.70796932 | 3.55618520 | 1.05804840 | 0.00003656 | 0.00023076 | Up |
| AL133370.1 | 1.18049419 | 0.45225954 | -1.38416810 | 0.00005301 | 0.00031610 | Down |
| ADAMTS9-AS1 | 1.01334921 | 0.30292546 | -1.74209668 | 0.00000000 | 0.00000000 | Down |
| SNHG18 | 6.72063319 | 2.57669539 | -1.38307517 | 0.00000000 | 0.00000000 | Down |
| LINC01082 | 4.04531483 | 1.95519690 | -1.04893808 | 0.00000005 | 0.00000080 | Down |
| AL645608.6 | 0.64749517 | 1.30626962 | 1.01251137 | 0.01492543 | 0.03369923 | Up |
| AC093001.1 | 11.41082587 | 28.07068219 | 1.29866091 | 0.00333755 | 0.00992078 | Up |
| AC105219.3 | 0.62322312 | 1.37628715 | 1.14296084 | 0.00000000 | 0.00000000 | Up |
| LINC01679 | 0.69559754 | 0.31514407 | -1.14224132 | 0.00000000 | 0.00000000 | Down |
| TPM1-AS | 1.33073208 | 0.66186940 | -1.00760166 | 0.00717332 | 0.01849462 | Down |
| LINC01866 | 0.89548337 | 2.55488179 | 1.51251800 | 0.01072163 | 0.02560546 | Up |
| MKX-AS1 | 0.64465751 | 0.27084647 | -1.25105762 | 0.00000000 | 0.00000000 | Down |
| LINC01926 | 4.09248956 | 1.74049646 | -1.23347985 | 0.00000000 | 0.00000000 | Down |
| LINC01612 | 0.19591856 | 1.00410576 | 2.35758527 | 0.00939539 | 0.02311979 | Up |
| OSTN-AS1 | 0.05692605 | 1.55823717 | 4.77468214 | 0.00030478 | 0.00138755 | Up |
| ADIRF-AS1 | 1.16561802 | 0.56138618 | -1.05402962 | 0.00000000 | 0.00000000 | Down |
| LINC02106 | 0.59258488 | 0.25490896 | -1.21703972 | 0.00000000 | 0.00000000 | Down |
| CARMN | 2.94898806 | 0.32527828 | -3.18047358 | 0.00000000 | 0.00000000 | Down |
| AC099850.4 | 0.60022937 | 1.47995507 | 1.30196756 | 0.00000004 | 0.00000072 | Up |
| AC011523.1 | 1.45484810 | 3.07828808 | 1.08125972 | 0.00000017 | 0.00000246 | Up |

**Supplement Table 2.** Clinical information for PCa patients sets in this study

| **Covariates** | **Total set (n=482)** | **Test set (n=240)** | **Train set (n=242)** | **P value** |
| --- | --- | --- | --- | --- |
| **Age** |  |  |  | 0.4789 |
| <65 | 321(66.6%) | 164(68.33%) | 157(64.88%) |  |
| >=65 | 161(33.4%) | 76(31.67%) | 85(35.12%) |  |
| **T stage** |  |  |  | 0.2965 |
| T1-T2 | 372(77.18%) | 179(74.58%) | 193(79.75%) |  |
| T3-T4 | 107(22.2%) | 60(25%) | 47(19.42%) |  |
| unknown | 3(0.62%) | 1(0.42%) | 2(0.83%) |  |
| **N stage** |  |  |  | 0.522 |
| N0 | 336(69.71%) | 170(70.83%) | 166(68.6%) |  |
| N1 | 75(15.56%) | 39(16.25%) | 36(14.88%) |  |
| unknown | 71(14.73%) | 31(12.92%) | 40(16.53%) |  |
| **M stage** |  |  |  | 0.8235 |
| M0 | 442(91.7%) | 219(91.25%) | 223(92.15%) |  |
| M1 | 3(0.62%) | 2(0.83%) | 1(0.41%) |  |
| unknown | 37(7.68%) | 19(7.92%) | 18(7.44%) |  |
| **GS** |  |  |  | 0.5638 |
| GS<8 | 290(60.17%) | 148(61.67%) | 142(58.68%) |  |
| GS≥8 | 192(39.83%) | 92(38.33%) | 100(41.32%) |  |

Abbreviations: PCa: Prostate cancer; GS: Gleason score
